# Mixtures of PRR Ligands Partly Mimic the Immunomodulatory Response of *γ*i *Staphylococcus aureus*, Enhancing Osteogenic Differentiation of Human Mesenchymal Stromal Cells

**DOI:** 10.1155/sci/1445520

**Published:** 2025-05-25

**Authors:** Paree Khokhani, Kelly Warmink, Moyo Kruyt, Harrie Weinans, Debby Gawlitta

**Affiliations:** ^1^Department of Orthopedics, University Medical Centre Utrecht, Utrecht, Netherlands; ^2^Department of Developmental BioEngineering, University of Twente, Enschede, Netherlands; ^3^Department of Biomechanical Engineering (3ME), Technical University Delft, Delft, Netherlands; ^4^Department of Oral and Maxillofacial Reconstruction Surgery and Special Dental Care, University Medical Centre Utrecht, Utrecht, Netherlands

**Keywords:** bone regeneration, immune modulation, inflammation, pathogen recognition molecular patterns, PRR ligands, *Staphylococcus aureus*

## Abstract

Recent evidence indicates the potential of gamma-irradiated (*γ*i) *Staphylococcus aureus* to be used as an osteo-immunomodulator for bone regeneration. This study aims at characterizing the inflammatory milieu caused by the stimulation of *γ*i *S. aureus* in immune cells and investigates its effects on MSC osteogenic differentiation. Furthermore, we aimed to recreate the immune-modulatory response exhibited by *γ*i *S. aureus* by using a mixture of various synthetic pathogen recognition receptor (PRR) ligands consisting of TLR2, TLR8, TLR9, and NOD2 agonists. Human peripheral blood mononuclear cells (hPBMCs), isolated from healthy human donors, were exposed to *γ*i *S. aureus* or seven different ligand mixtures. After 24 h, the conditioned medium (CM) from the hPBMCs was collected and its effects on hMSC osteogenic differentiation were investigated by assessing alkaline phosphatase (ALP) activity and matrix mineralization. The hPBMCs and their CM were also analyzed by bulk RNA sequencing and for cytokine secretion. CM from the *γ*i *S. aureus* and the mixture consisting of Pam3CSK4, C-class CpG oligodeoxynucleotide (CpG ODN C), and murabutide targeting TLR2, TLR9, and NOD2 showed a fivefold increase in ALP and matrix mineralization in a donor-dependent manner. These effects were due to the upregulation of inflammatory signaling pathways, which led to an increase in cytokines and chemokines TNF, interleukin (IL)-6, IFN-*γ*, IL-1*α*, CXCL10, CCL18, CCL17, CXCL1, and CCL5. Upregulation of genes like BMP2R, BMP6R, BGLAP, and others contributed to the upregulation of osteogenic pathways in the hPBMCs stimulated with *γ*i *S. aureus* and the aforementioned mix. Thus, formulations with mixtures of PRR ligands may serve as immune-modulatory osteogenesis-enhancing agents.

## 1. Introduction

New strategies to enhance off-the-shelf bone substitutes, especially biphasic calcium phosphates (BCPs), are of great interest, considering their noninferiority to the existing autologous bone grafting to treat critical size defects, spinal fusion, and fracture nonunions [1, 2]. BCPs are an attractive alternative to the current gold standard autografts as they are biocompatible, demonstrate osteoconductivity, have an unlimited supply, and are easy to sterilize. However, autografts and BCPs only achieve a 58%–93% spinal fusion rate [2–5 ]. Adding cells or growth factors to these bone substitutes to boost their performance is explored extensively [6, 7]. With increasing evidence of the immune system's role in bone healing and repair, the use of immune-modulatory factors to enhance bone substitutes holds excellent potential [8–10].

Generally, a balanced early inflammatory phase provides the vital microenvironment that coordinates osteogenic and angiogenic processes that are needed for optimal bone healing [11, 12]. Pathological conditions such as osteomyelitis and heterotopic ossification, which involve the activation of inflammatory pathways leading to bone formation, can provide cues in identifying the underlying pro-osteogenic and angiogenic factors [13, 14]. For instance, local responses to bacterial infections have been associated with new bone formation in heterotopic ossifications due to increased inflammation, indicating that bacterial antigens may act as osteo-immunomodulators [15, 16]. Recently, we showed that gamma-irradiated (*γ*i) *Staphylococcus aureus* or its cell wall fragments induced bone formation in several rabbit models [17, 18]. The mechanism behind this phenomenon is assumed to be the sterile inflammatory microenvironment created by *γ*i *S. aureus* or the cell wall components that might be beneficial for bone formation; however, the nature and strength of inflammatory stimuli may distinctly influence the process of bone formation [19–21 ]. Furthermore, it is known that genetic and environmental factors shape immune cell responsiveness, which may in turn affect MSC-mediated osteogenesis [22, 23]. Hence, elucidating the mechanism through which *γ*i *S. aureus* and the immune cell secretome in response to *γ*i *S. aureus* stimulation affect bone formation is of great interest to define the inflammatory milieu that promotes bone regeneration.

The host immune system recognizes *S. aureus* by identifying the pathogen-associated molecular patterns (PAMPs) via the pathogen recognition receptors (PRRs) and inflammasome signaling, which lead to the release of various proinflammatory cytokines and antimicrobial peptides [24, 25]. The primary PRR in *S. aureus* detection is the plasma membrane-bound TLR2, which is activated upon sensing bacterial lipoteichoic acid (LTA), or synthetic ligand Pam3CysSerLys4 (Pam3CSK4), to form heterodimers TLR1/TLR2 and TLR2/TLR6 [26, 27]. In addition, small peptidoglycan fragments of *S. aureus*, also synthetically known as murabutide, induce cytosolic NOD-2 activation, resulting in the induction of various cytokines like interleukin (IL)-1*β*, IL-6, and antimicrobial peptides [28–30 ]. Upon degradation of *S. aureus* in the phagosome, the classical induction of inflammatory cytokines and interferons is triggered due to the detection of CpG motifs by TLR9 [31, 32], via nuclear factor kappa beta (NF-*κ*B), mitogen-activated protein kinase (MAPK), type I interferons, and inflammasome signaling pathways [33]. To sum up, a synergistic crosstalk between the PRRs may be the reason for a controlled and yet specific immune response observed in *S. aureus* exposure.

Mixing different PRR ligands involved in the recognition of *S. aureus* via the immune system may prove to be an effective alternative for enhanced bone healing. PRR ligands are explored extensively for their ability to stimulate and enhance the host immune response. For example, they are currently clinically tested as vaccine adjuvants for cancer therapies and infectious diseases [34, 35]. Recently, we also examined the effects of individual PRR ligands on osteogenic differentiation of human mesenchymal stromal cells (hMSCs) in vitro. Intracellular nucleic acid–based PRR ligands enhanced osteogenic differentiation when added directly or through conditioned medium (CM) from stimulated immune cells [36, 37]. Considering this, PRR ligands offer a better-defined and more stable alternative to overcome the drawbacks like off-target side effects and batch-to-batch inconsistencies of *γ*i *S. aureus* for orthopedic applications.

In this study, we evaluated the immunomodulatory effects and the underlying mechanism of *γ*i *S. aureus* on osteogenic differentiation of hMSCs. Further, we aimed to mimic the effect of *γ*i *S. aureus* on osteogenic differentiation by stimulating hMSCs with several mixtures of PRR ligands, as these have the potential to provide a better defined and safer alternative compared to *γ*i *S. aureus* for orthopedic applications. Additionally, hMSCs were indirectly stimulated via conditioned media from human peripheral blood mononuclear cells (hPBMCs) exposed to *γ*i *S. aureus* or ligands to mimic the in vivo situation where peripheral blood is exposed before hMSCs arrive to the scene. To achieve this, we characterized hMSCs and hPBMCs stimulated with either *γ*i *S. aureus* or mixtures to gain insight into the signaling pathways, secreted cytokines, and growth factors with the ultimate goals to improve bone (re)generation and to use a specific set of PRR ligands instead of *γ*i *S. aureus*.

## 2. Materials and Methods

### 2.1. Study Design

The osteogenic potential of *γ*i *S. aureus* and mixtures containing synthetic PRR ligands mimicking *S. aureus* was investigated by adding them either directly (direct stimulation) or via the CM obtained from stimulation of immune cells (indirect stimulation) to the bone marrow-derived hMSCs. The mixtures were based on the PRRs involved in the initial recognition of *S. aureus* mentioned in the literature [24] (Table 1).

For indirect stimulation (immune-mediated stimulation), hPBMCs were isolated from peripheral blood obtained from healthy donors (*n* = 9). The hPBMCs were seeded in a 24-well plates and cultured in the presence/absence of *γ*i *S. aureus* or mixtures of PRR ligands (Table 1). The unstimulated hPBMCs served as the control. After 24 h, the hPBMCs were prepared for bulk RNA sequencing experiments. The pooled supernatant from the hPBMCs donors was centrifuged at 1500 × *g* for 5 min to obtain cell-deprived CM that was stored at −80°C. The CM was used to characterize its composition using a custom Luminex assay. Furthermore, the CM was added to the hMSC culture in the ratio of 1:4 (CM: osteogenic differentiation medium (ODM)); this ratio was chosen based on previous research [36]. The osteogenic differentiation of hMSCs was evaluated by assessing the secretion of alkaline phosphatase (ALP) on days 3, 7, 10, and 14 along with matrix mineralization on day 21.

### 2.2. Reagents

*Staphylococcus aureus* (Wood 46) was cultured to mid-log phase in LB liquid medium. Freshly cultured bacteria were then subjected to 25 Gk*γ* radiation. The absence of any viable bacteria was confirmed by plate cultures. The killed bacteria suspensions were stored at −80°C in phosphate-buffered saline (PBS) solution (Gibco, Thermo Fisher Scientific, United States) with 20% (*v*/*v*) glycerol. The bacteria were washed thoroughly with PBS (300 g for 3 min) before use. *γi S. aureus* was used in the concentrations of 10^7^ units/mL (high), 10^6^ units/mL (medium), and 10^5^ units/mL (low) for the experiments.

C-class CpG oligodeoxynucleotide (CpG ODN C; M362; 1 µg/mL), resiquimod (R848; 10 µg/mL), Pam3CSK4 (Pam3CysSerLys4; 10 µg/mL), CL429 (Pam2C-conjugated murabutide; 10 µg/mL), insoluble peptidoglycan (from *S. aureus*; 10 µg/mL), LTA (from *S. aureus*; 10 µg/mL), and murabutide (10 µg/mL) were purchased from InvivoGen, USA. These PRR ligands were mixed to form mixtures 1–7, of which the composition was based on PRRs involved in the recognition of *S. aureus*, as mentioned in the literature [24]. The concentration of each PRR ligand was chosen based on the manufacturer's datasheet.

### 2.3. Isolation and Culturing of hPBMCs

Human blood from healthy donors with ages ranging from 27 to 62 years (*n* = 9) was obtained with the approval of the local medical ethical committee (University Medical Center Utrecht, Utrecht, The Netherlands) under the protocol METC 07-125/C and written consent of the participants.

Peripheral mononuclear cells (hPBMCs) were isolated using the density gradient centrifugation method. Briefly, the heparinized blood was diluted with PBS in a ratio of 1:1 and layered on top of the 15 mL density gradient medium (Ficollpaque Plus, GE Healthcare, United States) into SepMate isolation tubes (Stem Cell Technologies, Germany). These tubes were then centrifuged at 1200 × *g* for 10 min with brakes on at room temperature. The supernatant containing the serum and cells was quickly poured off in a new 50 mL Falcon tube and centrifuged at 300 × *g* for 8 min at room temperature. After centrifugation, hPBMCs were then suspended in a culture medium consisting of RPMI-1640 glutamax (Thermo Fisher Scientific, United States) supplemented with 10% (*v*/*v*) heat-inactivated fetal bovine serum (FBS, Biowest, United States) and 100 U/mL penicillin and 100 mg/mL streptomycin (Gibco, Thermo Fisher Scientific, United States).

To produce 1 mL CM, hPBMCs from individual donors were seeded in a 24-well plates at 500,000 cells/cm^2^ and cultured for 24 h at 37°C in a humidified atmosphere containing 5% CO_2_. Subsequently, the hPBMCs were stimulated with either *γi S. aureus* or one of the mixtures (Table 1). Unstimulated hPBMCs served as a control. After 24 h of culture, hPBMCs were stored in TRIzol reagent (Thermo Fisher Scientific, United States) for extraction of RNA while the CM was pooled from all donors per condition, centrifuged at 1500 × *g* for 5 min, and stored at −80°C until further use.

### 2.4. Isolation and Culturing of hMSCs

Bone marrow was harvested from the vertebrae or the iliac crest of female patients aged 15–62 years (*n* = 6) with the approval of the local medical ethical committee (University Medical Center Utrecht, Utrecht, The Netherlands) under biobank protocol 08/001K with broad consent. hMSCs were isolated and cryopreserved at passage 2 according to a standardized method that yields multipotent cells [38]. hMSCs were thawed and expanded in a standard expansion medium consisting of minimum essential medium (*α*-MEM, Gibco, Thermo Fisher Scientific, United States) supplemented with 10% (*v*/*v*) heat-inactivated FBS (Biowest, France) and 100 U/mL penicillin and 100 mg/mL streptomycin and 0.2 mM l-ascorbic acid-2-phosphate (Sigma–Aldrich, United States). The cells were plated at 70% confluency during subsequent passages up to a maximum of five passages. All cell cultures were performed at 37°C in a humidified atmosphere containing 5% CO_2_.

To test the effect of the *γ*i *S. aureus* and the mixtures on early and late osteogenic differentiation, hMSCs were seeded at a density of 15,000 cells/cm^2^ in a 96- or 24-well plates in technical duplicates and cultured in the expansion medium. Upon 100% confluency, culture continued with either expansion medium or osteogenic medium (supplemented with 10 mM *β*-glycerophosphate and 10 nM dexamethasone, both from Sigma–Aldrich, United States) and in the presence/absence of the CM or by adding the *γ*i *S. aureus* and the mixtures directly. hMSCs cultured in the osteogenic medium alone and in osteogenic medium containing 25% CM obtained from unstimulated hPBMCs were controls. The medium was refreshed every 3 days until day 14 for early analyses and day 21 for late osteogenic differentiation analyses.

### 2.5. hMSC Osteogenic Differentiation Assays

#### 2.5.1. ALP Secretion Quantification

For quantification of ALP, cells were cultured in the ODM containing 10% FCS (Biowest, France). On days 3, 7, 10, and 14, cells were lysed in 0.2% (*v*/*v*) Triton X-100/TBS for 30 min. ALP activity was measured by the conversion of the p-nitrophenyl phosphate liquid substrate system (pH = 9.6; SigmaFast p-nitrophenyl phosphate tablets, Sigma–Aldrich). The cell lysate was also used to determine the DNA content with the Quant-It PicoGreen kit (Invitrogen), according to the manufacturer's instructions. The ALP/DNA ratio was normalized to the ratio of the controls that were treated with CM from unstimulated hPBMCs or osteogenic medium alone. The concentration of *γ*i *S. aureus* and mixtures that yielded highest ALP activity upon indirect stimulation was chosen for further experiments (Figure S1, the ones highlighted in red boxes).

#### 2.5.2. Matrix Mineralization Assay

hMSCs were cultured in an osteogenic medium containing 10% FCS (Gibco, Thermo Fisher Scientific, United States) to assess and quantify the matrix mineralization after 21 days. Samples were incubated with 0.2% Alizarin Red S (ARS) for 60 min (pH = 4.2, Sigma) and examined using light microscopy. In addition, Alizarin Red was extracted from the monolayer of the cells by incubating in 10% (*w*/*v*) cetyl pyridinium dissolved in 10 mM sodium diphosphate buffer solution (pH = 7.2) (Sigma–Aldrich) for 60 min. Absorbance was measured at 595 nm and corrected at 655 nm. The amount of calcium deposited in each well (experiments done in duplicates) was quantified using the standard curve obtained by dissolving a known concentration of ARS and considering 2 mol of Ca^2+^/mol of dye in the solution.

### 2.6. Bulk RNA Sequencing and Analysis

Extraction of total RNA from hPBMCs stimulated with either *γ*i *S. aureus* or the mixtures for 24 h was carried out using the RNAeasy micro kit (Qiagen, Germany) according to the manufacturer's instructions. The RNA concentration and quality were assessed using automated electrophoresis (4200 TapeStation system, Agilent). Bulk RNA sequencing was performed by single cell discoveries (Utrecht, The Netherlands) using the CEL-seq 2 protocol with a sequencing depth of 10 million reads per sample. R data programming version 3.7 was used to analyze the data. The count normalization and differential gene expression were performed using the DESeq2 package v3.15 [39]. The Log2 fold change of different genes between the experimental groups (i.e., unstimulated control versus *γ*i *S. aureus*, control vs. Mix 1, control vs. Mix 4, control vs. Mix 5) was determined. Log-fold change shrinkage was applied using the lfcShrink function with the adaptive student's *t* prior shrinkage estimator to reduce the number of false positives [40]. This fold change was used in all downstream analysis. A Wald test statistic was used to estimate fold change significance and *p*-values were adjusted for multiple testing using the Benjamini–Hochberg method. Genes with a shrunken Log2 fold change above 1 and an adjusted *p*-value below 0.05 were considered differentially expressed. The top 100 differentially expressed genes between control and *γ*i *S. aureus* were visualized across all samples in a heatmap, expressing normalized transformed counts, scaled per gene to represent the deviation from the average. In addition, predetermined genes of interest were visualized similarly in heatmaps and/or gene expression plots by plotting the normalized transformed counts per condition.

The R package clusterProfiler (v.4.4.4) was used to perform gene set enrichment analysis (GSEA). The analysis was performed using gseGO to assess enrichment of Gene Ontology (GO) terms, with org.Hs.eg.db as the organism database. A *p*-value below 0.05 was considered a significant enrichment, all *p*-values were corrected for multiple testing using the Benjamini–Hochberg method. Dot plot visualizations were generated to display the most significant gene sets, categorized by activation or suppression, as well as specific GO terms of interest related to bone development and inflammation.

### 2.7. Characterization of CM Composition Using Luminex Assay

The CM was characterized for its protein composition using a human 36-target multiplex array (IL1RA, IL-1*α*, IL-1*β*, IL-6, IL-10, IL-17, IL-17F, TNF*α*, IFN*α*, IFN*β*, IFN*γ*, TGF*α*, MCP1, MIP1*α*, MIP1*β*, RANTES, MCP3, MCP2, CCL17, CCL18, GRO1a, CXCL5, IL8, MIG, IP10, SDF1*α*, OPG, OPN, SerpinF2, SerpinG1, FGFbasic, VEGF, MMP9, TREM1, IL1R1, and THBS1). This experiment was performed at the multiplex core facility (MPCF), University Medical Center, Utrecht, The Netherlands. The data were normalized to the unstimulated hPBMCs (control) and presented as Log2-change in a heatmap. The values that were out of range (OOR) were shown in the heatmap as OOR: OOR > (above the highest concentration of the standard curve) or OOR < (below the lowest concentration of the standard curve).

### 2.8. Statistical Analysis

The normal Gaussian distribution of the data was tested using the Shapiro–Wilk normality test. Repeated measures ANOVA with Dunnett's post hoc correction for multiple comparisons was performed using SPSS (V24, IBM, USA). *p*-value  < 0.05 was used as a threshold for significance. All data are presented as mean ± standard deviation with sample sizes mentioned in the figure legends. The statistical analysis for the bulk RNA seq data is detailed under the respective paragraph.

## 3. Results

### 3.1. *γ*i *Staphylococcus aureus* and Mixtures Show Different Patterns of PRR Activation

**Figure 1:**
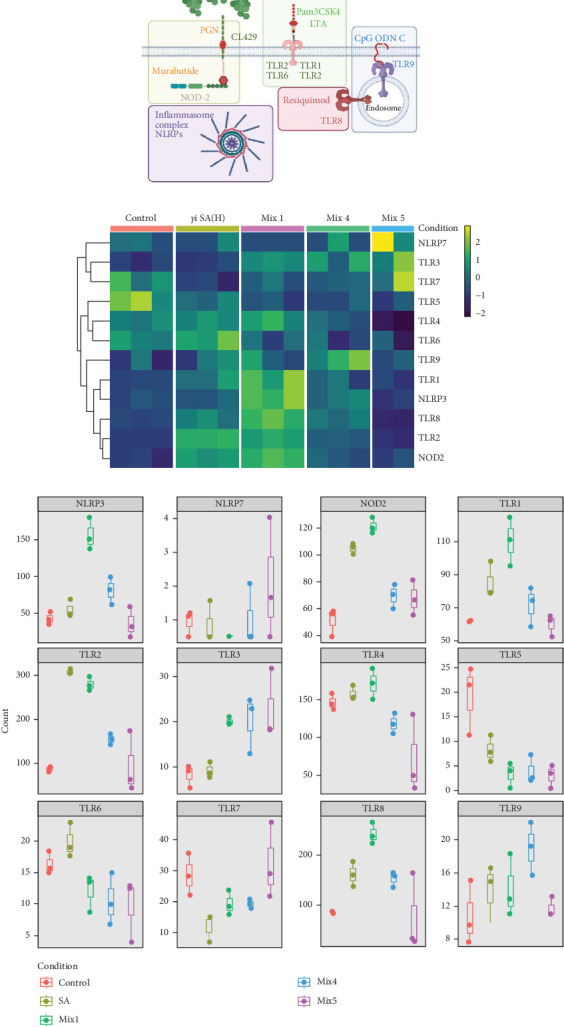
Effect of *γ*i *Staphylococcus aureus* and mixtures on pathogen recognition receptor expression at the gene level in pooled hPBMCs (*n* = 9). (a) Illustration showing the PRRs involved in *Staphylococcus aureus* recognition based on literature [28]. Colored boxes correspond to colors in Table 1. (b) Heatmap showing the log2 normalized and scaled of the absolute gene count between groups. Each box depicts the technical replicates of the conditions. (c) Absolute gene count of the PRRs for all groups. Data are shown as box and whisker plots. The colored dots indicate the technical replicates used per condition.

The gene expression in hPBMCs stimulated with *γ*i *S. aureus* showed a significant upregulation in TLR1, TLR2, TLR6, TLR8, TLR9, and NOD2 expression along with a downregulation in TLR5 expression, as compared to the nonstimulated hPBMC control (Figure 1) [24 ]. Mix 1 shows upregulation in PRR expression similar to *γ*i *S. aureus* except for NLRP3 and TLR6 expressions. Mix 4 and Mix 5, on the other hand, show a downregulation in NOD2, TLR1, TLR2, TLR4, and TLR7 as compared to *γ*i *S. aureus*. Upregulation of TLR3 was observed in all mixtures and not in *γ*i *S. aureus* (Figure 1c). Based on these, Mix 1 mimics *γ*i *S. aureus* most closely in terms of PRR expression (Figure 1b).

### 3.2. CM From *γ*i *Staphylococcus aureus* and Mixtures of PRR Ligands Enhance Osteogenic Differentiation of hMSCs, While Direct Stimulation Does not

**Figure 2:**
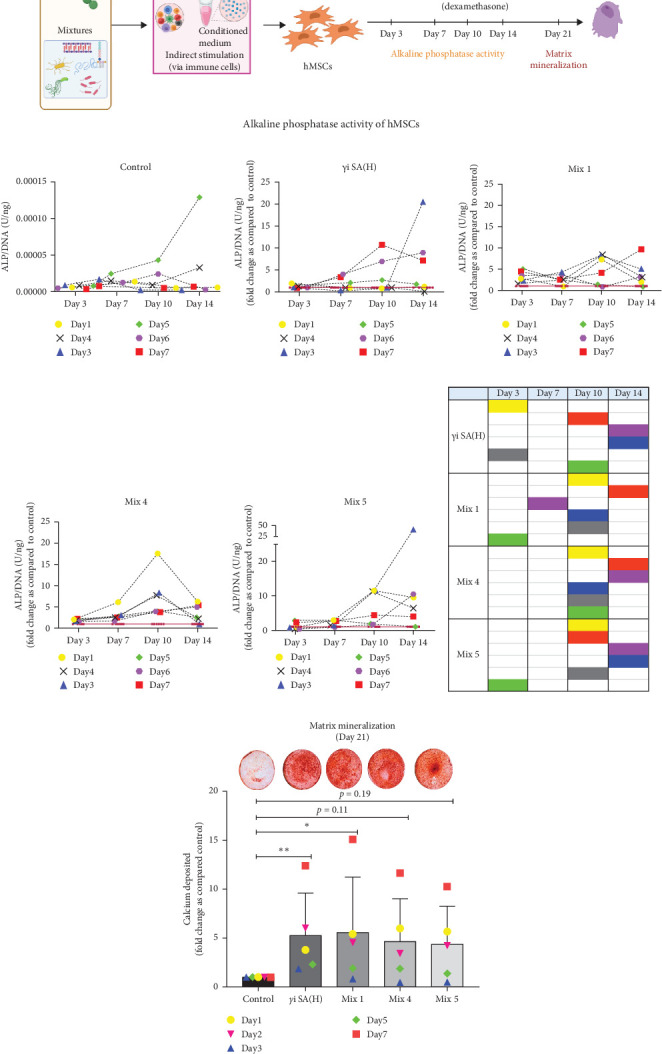
Indirect effect of *γ*i *Staphylococcus aureus* and mixtures on the hMSC osteogenic differentiation. (a) Study design setup. *γ*i *Staphylococcus aureus* and mixtures were added to hPBMCs (*n* = 9) for 24 h. The conditioned medium obtained from the stimulation was pooled and used to stimulate hMSCs (*n* = 6) into osteogenic differentiation. The CM was added in the ratio of 1:4 (CM: ODM). (b) Alkaline phosphatase activity was measured at days 3, 7, 10, and 14 and normalized to DNA content for the controls containing CM obtained from unstimulated hPBMCs. (c) The fold change of the normalized ALP activity to the controls is shown for *γ*i *Staphylococcus aureus*, (d) Mix 1, (e) Mix 4, and (f) Mix 5. The data are presented as the mean of technical duplicates performed per donor for six individual donors. Significance was tested using repeated-measures ANOVA with Sidak's post hoc test for multiple comparisons. (g) Tabular format showing the upregulation of six hMSC donors (in colored cells) for ALP activity between groups. (h) Alizarin Red S staining was performed after hMSCs (*n* = 5) were stimulated with CM obtained from *γ*i *Staphylococcus aureus* and mixtures Mix 1, Mix 4, and Mix 5 stimulated hPBMCs (*n* = 9). The stained images (circular wells) show the calcium deposition in red for all groups (representative for five MSC donors). The total amount of calcium deposited per well was quantified and normalized to the control. Results show the mean + standard deviation for five MSC donors. Significance was tested using one-way ANOVA with Sidak's post hoc test for multiple comparisons. *⁣*^*∗*^*p* < 0.05, *⁣*^*∗∗*^*p* < 0.01, and *⁣*^*∗∗∗*^*p* < 0.001.

hMSCs stimulated with a high concentration of *γ*i *S. aureus* CM showed increased ALP activity at all time points as compared to the control group in 3/6 hMSC donors (Figure 2c). A similar trend was observed with conditioned media obtained from hPBMCs stimulated with mixtures, mainly Mix 1, Mix 4, and Mix 5 in 5/6 hMSC donors (Figure 2), while no effect was observed when individual PRR ligands were used (Figure S2). The timing of the effect in all the groups was donor-dependent (Figure 2g). The direct stimulation with *γ*i *S. aureus* and mixtures did not show an increase in the ALP activity (Figure S3). In addition, no effect on ALP activity was observed when hMSCs were cultured in normal expansion medium, indicating a synergistic response of *γ*i *S. aureus* and mixtures Mix 1, Mix 4, and Mix 5 with osteogenic medium (Figure S4). In agreement with the early osteogenic marker ALP activity, hMSCs stimulated indirectly with a high concentration of *γ*i *S. aureus* CM or with Mix 1 CM showed a fivefold increase compared to the control for matrix mineralization, a late marker for osteogenic differentiation (Figure 2h).

### 3.3. Differential Gene Expression and Gene Set Enrichment Analysis for *γ*i *Staphylococcus aureus* and Mixtures

**Figure 3:**
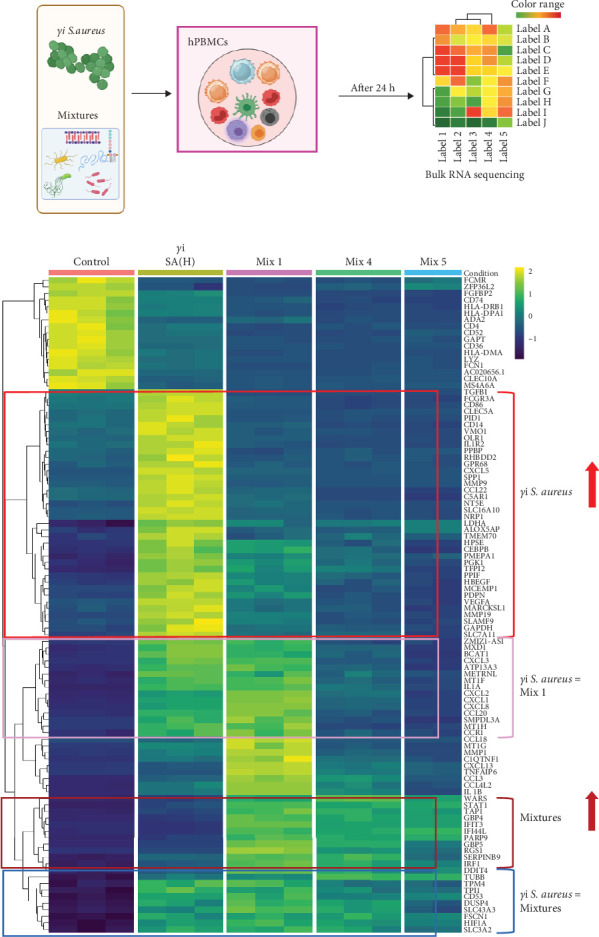
Differential gene expression analysis. (a) Illustration of the assay setup. hPBMCs (*n* = 9) were stimulated with either *γ*i *Staphylococcus aureus* or mixtures for 24 h. RNA was isolated and processed for bulk RNA sequencing. RNA pooled from unstimulated hPBMCs (*n* = 9) served as the control. (b) A heatmap is showing gene expression of all groups of the top 100 most differentially regulated genes between control and *γ*i *Staphylococcus aureus*. Color scale indicates normalized transformed gene expression, scaled per gene to represent deviation from the average. The colored boxes highlight the patterns observed in the genes among all the groups.

We identified the top 100 differentially expressed genes (Figure 3) between *γ*i *S. aureus* and the control group and compared their gene expression across all experimental groups (Figure 3b). Of these 100 genes, 38 genes were upregulated in the *γ*i *S. aureus* group as compared to control, while showing low expression for all the mixtures (Figure 3b: highlighted in red). Amongst these genes are transcription factors for various inflammatory markers like CXCL5, CCL22, IL-1R1, and growth factors like VEGF, TGF*β*1, and degrading enzyme MMP9, and encoder of bone matrix protein SPP1, which are known to play a role in the fracture healing process. Fourteen genes showed similar patterns between *γ*i *S. aureus* and Mix 1 (Figure 3b: highlighted in pink). These were mostly chemokines and pro-inflammatory cytokines, like IL1A and several CXCLs. Further, 12 genes showed a similar pattern between the three mixtures, all showing a high expression (Figure 3b: highlighted in brown), whereas the control and *γ*i *S. aureus* group showed low expression levels. Last, nine genes were found to show a similar pattern between *γ*i *S. aureus* and all mixtures as compared to the control (Figure 3b: highlighted in blue). Most notably, HIF-1*α* is upregulated, which is an important factor in vascular recruitment and subsequent bone formation [41]. All together indicating that Mix 1 shows the most overlap in the response to *γ*i *S. aureus*.

**Figure 4:**
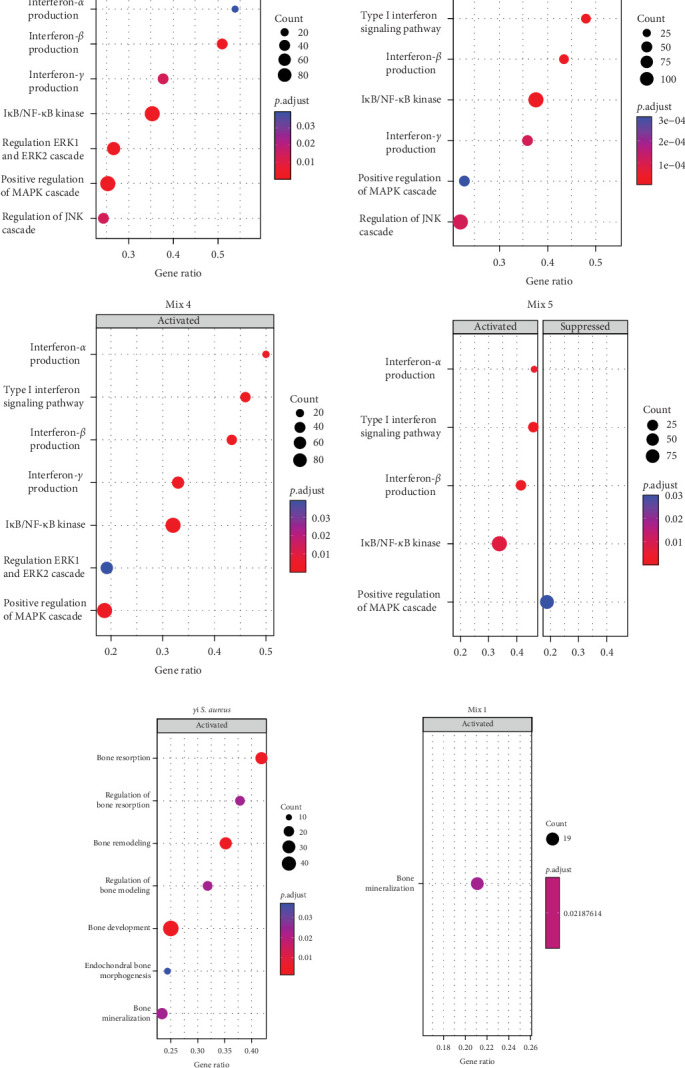
The gene set enrichment analysis (GSEA), comparing Gene Ontology term enrichment for *γ*i *Staphylococcus aureus* and mixtures (Mix 1, Mix 4, and Mix 5) stimulated hPBMCs compared to unstimulated control. (a) Bubble plot showing the activation of inflammatory signaling pathways in all groups as compared to the control. (b) Bubble plot showing the activation of bone development and bone remodeling pathways in all conditions as compared to the control. Mix 1 was the only mixture to show an upregulation of bone related pathways. The number of the genes differentially expressed in a pathway is displayed using the size of the circle, GeneRatio displays the differentially expressed genes as a proportion of all genes in the pathway. The significance is shown as a color gradient (scale bar).

To understand the biological processes and potential mechanisms involved in the immunomodulatory responses of the *γ*i *S. aureus* and the mixtures, we performed gene set enrichment analyses for *γ*i *S. aureus*, Mix 1, Mix 4, and Mix 5 compared to control on an a priori determined set of genes relating to bone and inflammatory pathways (Figure 4). The top 10 GO terms upregulated for the *γ*i *S. aureus*, Mix 1, Mix 4, and Mix 5 also showed a similar pattern (Figure S5). Especially *γ*i *S. aureus*, Mix 1, and Mix 4 stimulation in hPBMCs resulted in the significant upregulation of mainly pro-inflammatory pathways of the NF-*κ*B, ERK, and MAPK cascades, which are the known pathways involved in the secretion of various pro-inflammatory cytokines and chemokines as compared to the control. Mix 5, on the other hand, showed an upregulation of the NF-*κ*B pathway with a suppression of MAP kinase. The interferon signaling pathway resulting in the secretion of IFN-*α*, -*β*, and -*γ* was upregulated in all the conditions (Figure 4a). Interestingly, *γ*i *S. aureus* is the only group that showed an upregulation in the bone development and remodeling pathways. With one exception for Mix 1, which showed an upregulation in the bone mineralization pathway only (Figure 4b). Mix 4 and Mix 5 showed no enrichment of any bone-related pathway compared to the control indicating that Mix 1 most closely mimics *γ*i *S. aureus* response.

### 3.4. Protein Expression in *γ*i *Staphylococcus aureus* and Mixtures CM

**Figure 5:**
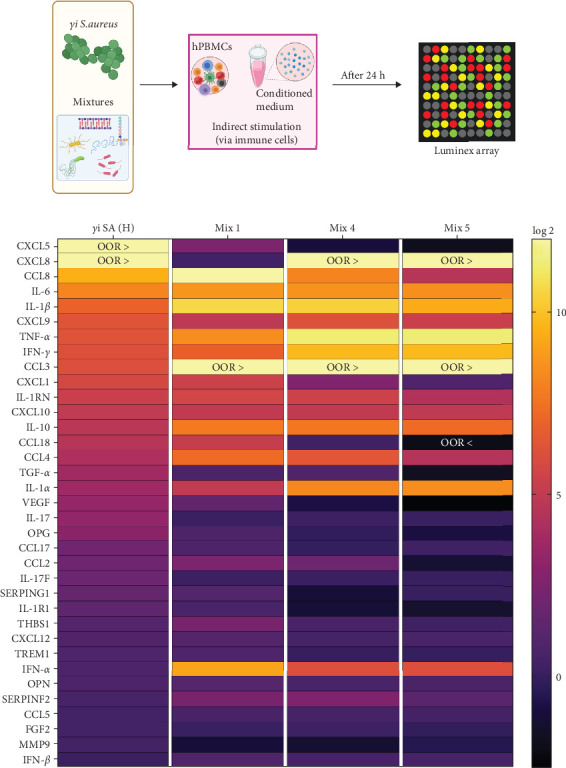
Characterization of proteins in CM. (a) hPBMCs (*n* = 9) were stimulated with either *γ*i *Staphylococcus aureus* or mixtures for 24 h. The conditioned medium obtained from the stimulation was pooled and used to characterize its composition using a Luminex assay. (b) Heatmap showing the Log2 fold change from control of different proteins. All the groups were normalized to the control and transformed using Log2. Samples that were above or below the detection limit were mentioned as out of range (OOR (above range OOR > and below range OOR <)).

On a protein level, *γ*i *S. aureus* CM showed an immunomodulatory pattern when compared to the CM of mixtures, since the concentrations of the proinflammatory cytokines were found to be higher than that in *γ*i *S. aureus* CM (Figure 5b). Significant upregulation was observed in proinflammatory cytokines and chemokines CCL8, IL-6, IL-1*β*, CXCL9, TNF-*α*, IFN-*γ*, CCL3, CXCL1, IL-1RN, CCL18, CCL4, and IL-1*α* in the Mix 1 CM as compared to *γ*i *S. aureus* CM (Figure S6). Mix 4 CM and Mix 5 CM also show a similar pattern to Mix 1, except for downregulation in CXCL1 and CCL18. Interestingly, *γ*i *S. aureus* CM showed an upregulation of cytokines IL-17, IL-17F, IL-1R1, chemokine CCL17, and growth factors TGF-*α*, VEGF, along with other factors like OPG, SERPING1 as compared to CM of the mixtures. Further, IFN-*α* was present in the higher concentrations in the mixtures CM as compared to that of *γ*i *S. aureus* CM.

## 4. Discussion

Harnessing bacterial antigens, especially those from *S. aureus* and their synthetic bacterial components known as PRR ligands, has gained attention to facilitate bone regeneration. Thus, understanding the mechanism and the inflammatory milieu instigated by *S. aureus* leading to bone formation is paramount to developing safe alternatives for patient treatment. To our knowledge, this is the first study to characterize the inflammatory milieu created by *γ*i *S. aureus* and SA-mimicking mixtures in immune cells and its positive effect on hMSC osteogenic differentiation in vitro.

In summary, we found that CM from cultured PBMCs that were stimulated with *γ*i *S. aureus* and mixtures of PRR ligands enhances osteogenic differentiation of hMSCs, while direct stimulation of hMSCs does not. The PRR mixture (Mix 1) mimicked the immunomodulatory response of *γ*i *S. aureus* in hPBMCs most closely based on the differential gene expression patterns in hPBMCs seen from bulk RNA sequencing as well as in the inflammatory cytokine pattern derived from the Luminex assay. The same Mix 1 also showed the highest osteogenic stimulation of hMSCs in terms of both the ALP activity and the matrix mineralization assay. Most strikingly, the GSEA revealed that *γ*i *S. aureus* stimulation upregulated genes like BMP6, BMP2, COL27A1, and BGLAP that contribute to the upregulation of bone development, bone remodeling, bone mineralization, and bone resorption pathways upon comparing to the predefined GO terms, whereas Mix 1 was the only mixture that showed an upregulation in bone mineralization pathway. These findings might explain previous observations of increased bone formation by bacterial antigens in various rabbit models [9, 17, 18].

We observed that the osteogenic effects of *γ*i *S. aureus* and PRR ligands are not directed to the MSCs (the precursors of osteoblasts) itself, but have an indirect role via the stimulated PBMC secretome. This conclusion is supported by our finding that CM from *γ*i *S. aureus* and PRR ligand mixtures enhanced osteogenic differentiation of hMSCs, whereas direct stimulation did not. It is important to note that previous studies have reported that direct stimulation with low-dose *S. aureus* exotoxin can enhance MSC osteogenesis [42, 43 ]. Differences in bacterial strain, dose, or experimental setup may explain this discrepancy.

On further characterization of the inflammatory milieu in hPBMCs CM, we found various cytokines like TNF-*α*, IL-6, IFN-*γ*, IL-1*α*, IL-1RN, IL-1R1, and chemokines CXCL10, CCL18, CCL17, CXCL1, CCL5, and factors osteoprotegerin, SERPING1. These were present in response to *γ*i *S. aureus* and the PRR ligands (in particular from Mix 1), which corresponds with activation of inflammatory signaling pathways NF-*κ*B, MAPK, ERK, and IFN type I upon comparing to the predefined GO terms as seen in the GSEA (Figures 4 and 5). The role of these proinflammatory cytokines in bone regeneration has been explored extensively. For example, TNF, IL-17, IL-1*β*, and IL-6 were shown to enhance MSC osteogenic differentiation 44–46]. Furthermore, the osteogenic potential of hPBMCs CM could also be explained by factors that were not investigated in this study, such as extracellular vesicles, which may contain immunomodulatory molecules, lipids, RNAs, and proteins and are known to play an important role in cell–cell communication [47].

Interestingly, only hPBMCs stimulated with *γ*i *S. aureus* showed an upregulation in genes that contribute to the upregulation of bone development, bone mineralization, bone resorption, and bone remodeling pathways as compared to the predefined gene sets, indicating that *γ*i *S. aureus* stimulation elicits a more comprehensive response in hPBMCs compared to PRR ligand mixtures (Figure 4). Nevertheless, the observed effect on matrix mineralization for CM of *γ*i *S. aureus* and the three mixtures of PRR ligands, especially Mix 1, in hMSCs was quite similar. This suggests that the effect on osteogenic differentiation is primarily linked to the inflammatory pathways activated in hPBMCs upon stimulation with the PRR ligands.

This study aimed at mimicking *γ*i *S. aureus's* immunomodulatory response by using various mixtures of PRR ligands. In agreement to numerous studies investigating the initial recognition mechanism of *S. aureus* in immune cells, we found that *γ*i *S. aureus* upregulates the gene expression of TLR1, TLR2, TLR6, NOD2, TLR8, and TLR9 in human PBMCs as compared to control [24, 27, 28, 48]. We expected the chosen mixtures to show a similar upregulation pattern in recognizing receptors, but only Mixture 1 closely mimicked the stimulation with *γ*i *S. aureus*. Even though PRR ligands targeting TLR2 and NOD2, that is, LTA, CL429, and murabutide were included in Mix 4 and Mix 5, an upregulation in TLR2 and NOD2 receptor expression was not observed (Figure 1). Further, the differential gene expression in hPBMCs revealed that 14 genes including CXCL1, CXCL2, CXCL8, CXCL3, and CCL20 showed similar expression when stimulated with *γ*i *S. aureus* and Mix 1, indicating that Mix 1 most closely mimics *γ*i *S. aureus* (Figure 3). The differences in the response of the mixtures might be explained from the differences in the chemical structure and origin of different PRR ligands resulting in nonspecific binding and triggering of the target receptors [49, 50]. Mixture 1 contained Pam3CSK4 to activate TLR2, compared to CL429 in Mix 4, and LTA in Mix 5. CL429 is a Pam2CSK4 conjugated with murabutide designed to activate TLR2 and NOD2. However, CL429 does not form heterodimers with TLR1 and TLR6 receptors [51]. LTA requires a costimulatory molecule CD36 to activate TLR2 as compared to Pam3CSK4 [52]. These differences in the activation pattern of the receptors were shown to result in a delayed activation of NF-*κ*B and MAPK pathways in murine macrophages [53]. Since all three mixtures (1, 4, and 5) contained the same components for NOD2 and TLR9 activation, however, the components present in TLR2 activation was different among the mixtures. This implies that the choice of the PRR ligand will affect all inflammatory and osteogenic aspects of the bone healing milieu.

Despite the differences in the gene expression pattern of the PRR receptors involved in recognition of *S. aureus* and mixtures along with the differences in differential gene expression, the CM from all three mixtures of PRR ligands enhanced MSC osteogenic differentiation relative to the nonstimulated control. This was evident from the matrix mineralization at day 21 (Figure 2). This is likely the result of secretion of proinflammatory cytokines and chemokines in hPBMCs due to a synergistic activation of PRRs, leading to an upregulation of the signaling pathways NF-*κ*B, MAPK, and of interferons. This activation of these inflammatory pathways plays a role in bone formation and homeostasis. For example, the p38 MAPK signaling pathway is shown to regulate functions of osteoblasts and chondrocytes leading to its contribution in bone development phases both in in vitro studies as well as in genetically modified rat models [54, 55]. Since all three mixtures show increased levels for some of the proinflammatory cytokines as well as the activation of the inflammatory signaling pathways as compared to *γ*i *S. aureus*, it can be hypothesized that the mixtures possess immunomodulatory properties that can be harnessed for bone regeneration.

From analyzing the cytokine pattern (Figure 5), the composition of the CM of Mixture 1 most closely resembles the cytokine profile of *γ*i *S. aureus*. With these findings, we showed that mixtures of PRR ligands can indeed at least partially recapitulate the immunomodulatory and osteoimmunological actions of *γ*i *S. aureus* for bone regeneration. In addition to their safety advantages, the mixtures of PRR ligands offer a highly customizable approach. The combination of ligands and their concentrations could be further tailored to suit specific bone regeneration requirements, potentially offering the option to fine-tune the treatment for various bone-related conditions and injuries such as, for example, bone defect or nonunions. Due to their size and small molecular structure, they can be easily integrated with tissue engineering constructs, in prosthetic coatings and drug delivery platforms. For example, PRR ligands can be encapsulated in nanoparticles such as liposomes to facilitate controlled and sustained release [56, 57]. In addition, their stability makes them ideal for use as bone-enhancing agents. Furthermore, they may offer a cheaper alternative to recombinant protein-based treatment options such as BMP-2. They can be easily immobilized on the surface of scaffolds, such as bioactive glass, titanium, or ceramics, using additive manufacturing techniques [58–61 ]. This allows for a smooth integration of PRR ligands into bone regenerative practices.

While these findings hold great promise, further research is essential to optimize mixture composition and to validate the safety and efficacy of mixtures of PRR ligands in clinically relevant animal models before considering human clinical trials. Several factors need to be considered for successful translation to the clinical setting, including age-related differences in MSC osteogenic potential. It is known that MSCs from older donors often display reduced proliferation and osteogenic differentiation capacity, while their adipogenic potential increases, which may affect their responsiveness to immune-modulatory factors such as PRR ligand-stimulated PBMC secretome [62]. Continued investigation will help optimize the mixtures dosage and delivery for the most favorable bone regeneration outcomes, ensuring the successful translation of this novel approach into treatments for patients with bone fractures, bone defects, and degenerative bone diseases. Ultimately, mixtures of PRR ligands could represent a groundbreaking advancement in bone healing, ushering in a new era of safe and effective bone regeneration therapies.

## 5. Conclusion

In conclusion, mixtures of PRR ligands can partly mimic the immunomodulatory response of *γ*i *S. aureus* and enhance osteogenic differentiation of hMSCs via stimulation of PBMCs. The PRR mixture (Mix 1) consisting of Pam3CSK4, CpG ODN C, and murabutide most closely mimicked the immunomodulatory *γ*i *S. aureus* response in hPBMCs indicating that the complete response of *S. aureus* is still not fully understood and needs further investigation. Although all formulations of mixtures with PRR ligands possess the potential to improve bone regeneration, considerable additional research is required for optimization and validation of these compounds to clinically advance bone healing.

## Figures and Tables

**Table 1:** Overview of the *Staphylococcus aureus* mimicking mixtures (MIX 1–7) that were prepared using the synthetic pathogen recognition receptor (PRR) ligands. Also, their corresponding recognition receptors are shown in the top row.

Mixtures	Recognizing PRRs			
	NOD-2	TLR2	TLR8	TLR9
Mix 1	Murabutide	Pam3CSK4	—	CpG ODN C
Mix 2	Murabutide, Peptidoglycan	—	—	CpG ODN C
Mix 3	Murabutide	—	—	CpG ODN C
Mix 4	Murabutide	CL429	Resiquimod	CpG ODN C
Mix 5	Murabutide	LTA	Resiquimod	CpG ODN C
Mix 6	Murabutide, Peptidoglycan	—	—	CpG ODN C
Mix 7	Murabutide	CL429	—	CpG ODN C

Abbreviations: LTA, lipoteichoic acid; TLR, toll-like receptors.

## Data Availability

The data that support the findings of this study are available from the corresponding author upon reasonable request.
