# Supplementary material for: Mixtures of PRR Ligands Partly Mimic the Immunomodulatory Response of γi Staphylococcus aureus, Enhancing Osteogenic Differentiation of Human Mesenchymal Stromal Cells
Source: Stem Cells Int. 2025 May 25;2025:1445520. doi: 10.1155/sci/1445520 (PMC12127128; doi:10.1155/sci/1445520)
Supplement: Supporting Information — figures S1–S6. [file 1445520.f1.docx]

Supplementary Figure S1.


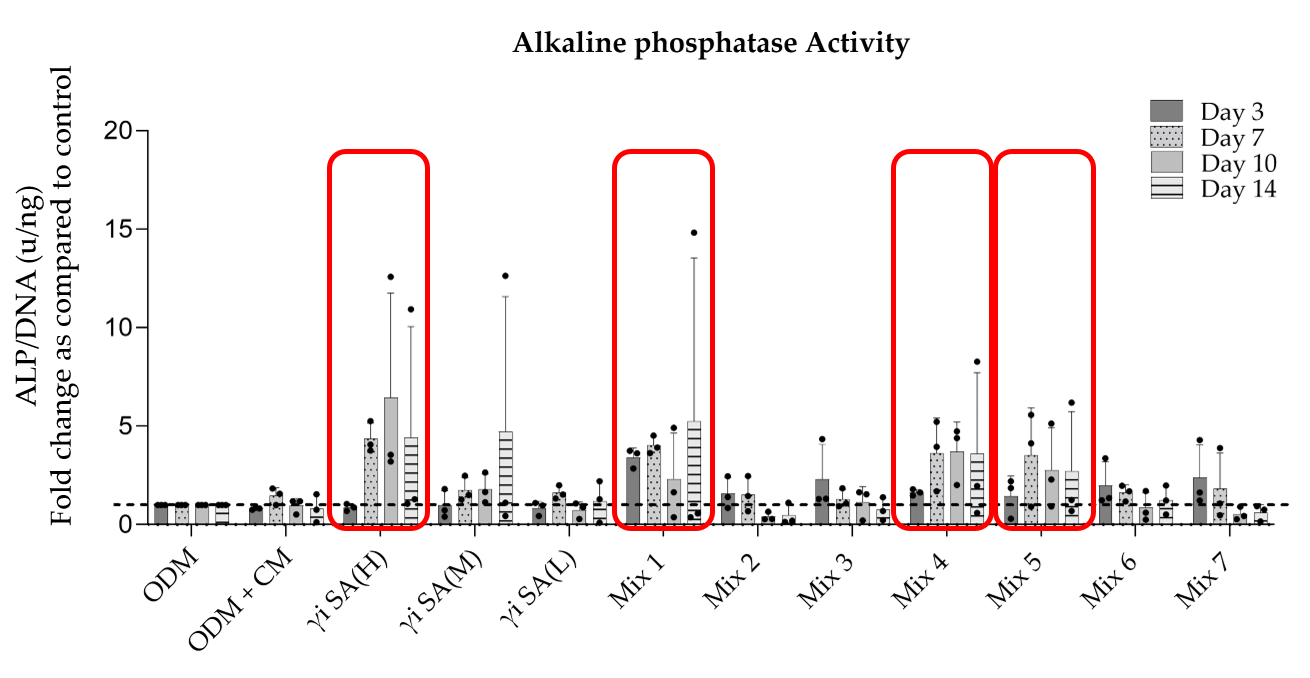
Supplementary Figure S1. Effect of indirect stimulation of γi *S. aureus* and mixtures on alkaline phosphatase levels in hMSC cultures, corrected for DNA on days 3,7,10, and 14. ALP/DNA ratios were normalized to the ratio of ODM cultured controls (dashed line). hMSC were cultured with conditioned medium obtained from hPBMCs (n = 6) stimulated with γi *S. aureus* in different concentrations and mixtures in the presence of osteogenic factor dexamethasone. The CM was added in the ratio of 1: 4 (CM: ODM). The graph represents the mean ± SD of technical triplicates performed per donor for three individual MSC donors. The groups γi *S. aureus* (H), Mix 1, Mix 4 and Mix 5 in (highlighted in the red boxes) were selected for further experiments.

Supplementary Figure S2.


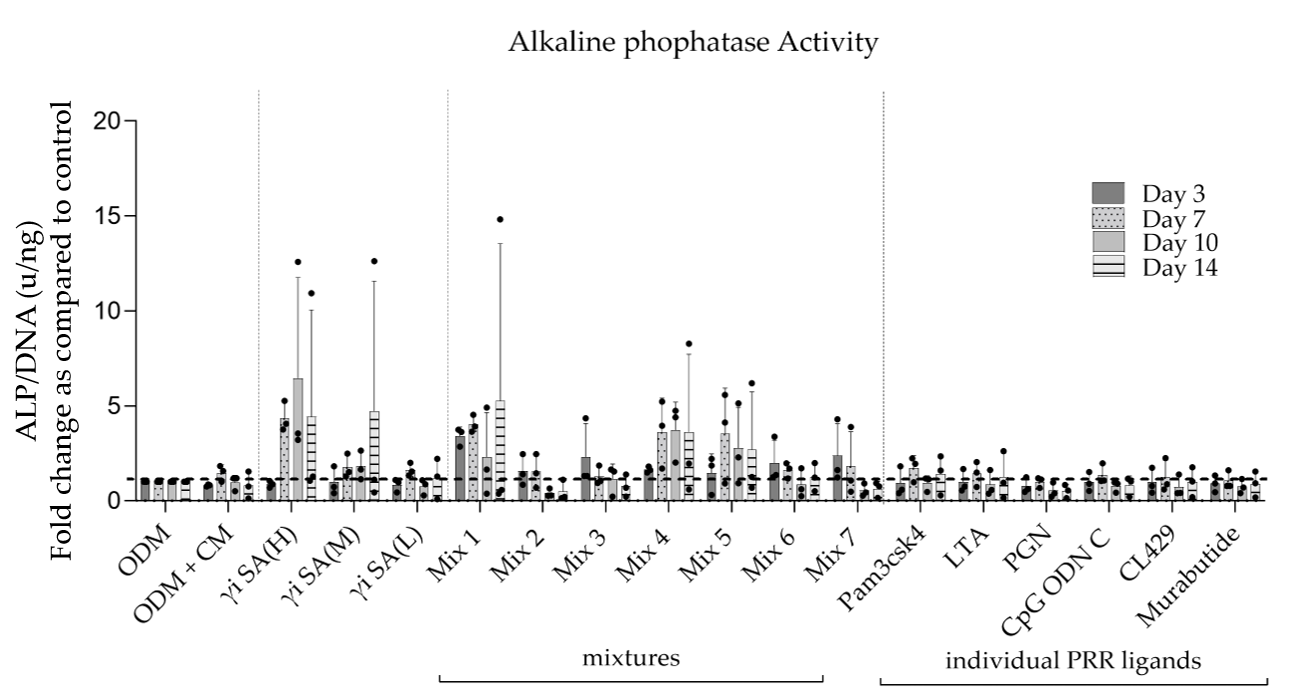


Supplementary Figure S2. Effect of indirect stimulation of γi *S. aureus* and mixtures on alkaline phosphatase levels in hMSC cultures, corrected for DNA on Days 3,7,10, and 14. hMSC were cultured with conditioned medium obtained from hPBMCs (n = 6) stimulated with γi *S. aureus* in 3 concentrations (H=high, M=middle, L=low), mixtures (1-7), and the individual PRR ligands in the presence of osteogenic factor dexamethasone. All data were normalized to the osteogenic medium control wells. The CM was added in the ratio of 1: 4 (CM: ODM). The graph represents the mean ± SD of technical triplicates performed per donor for three individual MSC donors (represented by dots).

Supplementary Figure S3.


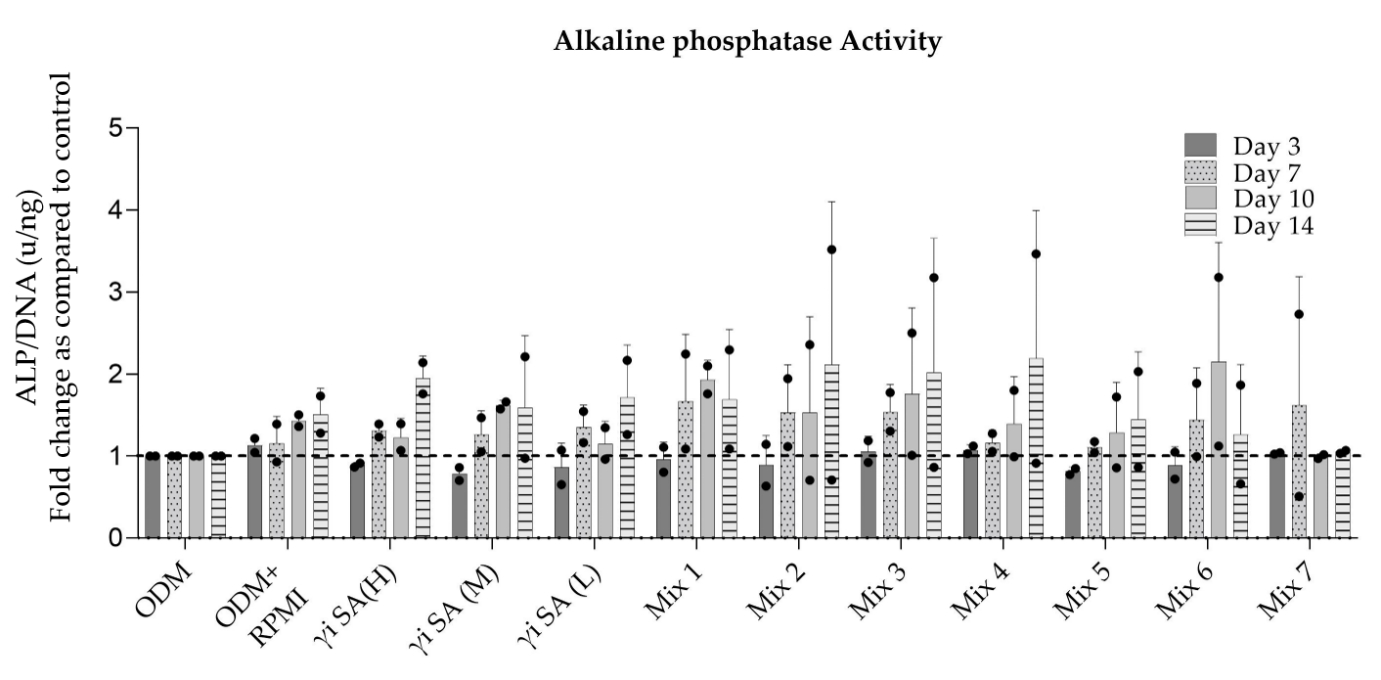


Supplementary Figure S3. Effect of direct stimulation of γi *S. aureus* and mixtures on alkaline phosphatase levels in hMSC cultures, corrected for DNA on Days 3,7,10, and 14. hMSC were cultured with conditioned medium obtained from hPBMCs (n = 6) stimulated with γi *S. aureus* in different concentrations and mixtures in the presence of osteogenic factor dexamethasone. All data were normalized to the osteogenic medium (ODM) control wells. The graph represents the mean ± SD of technical triplicates performed per donor for two individual MSC donors.

Supplementary Figure S4.


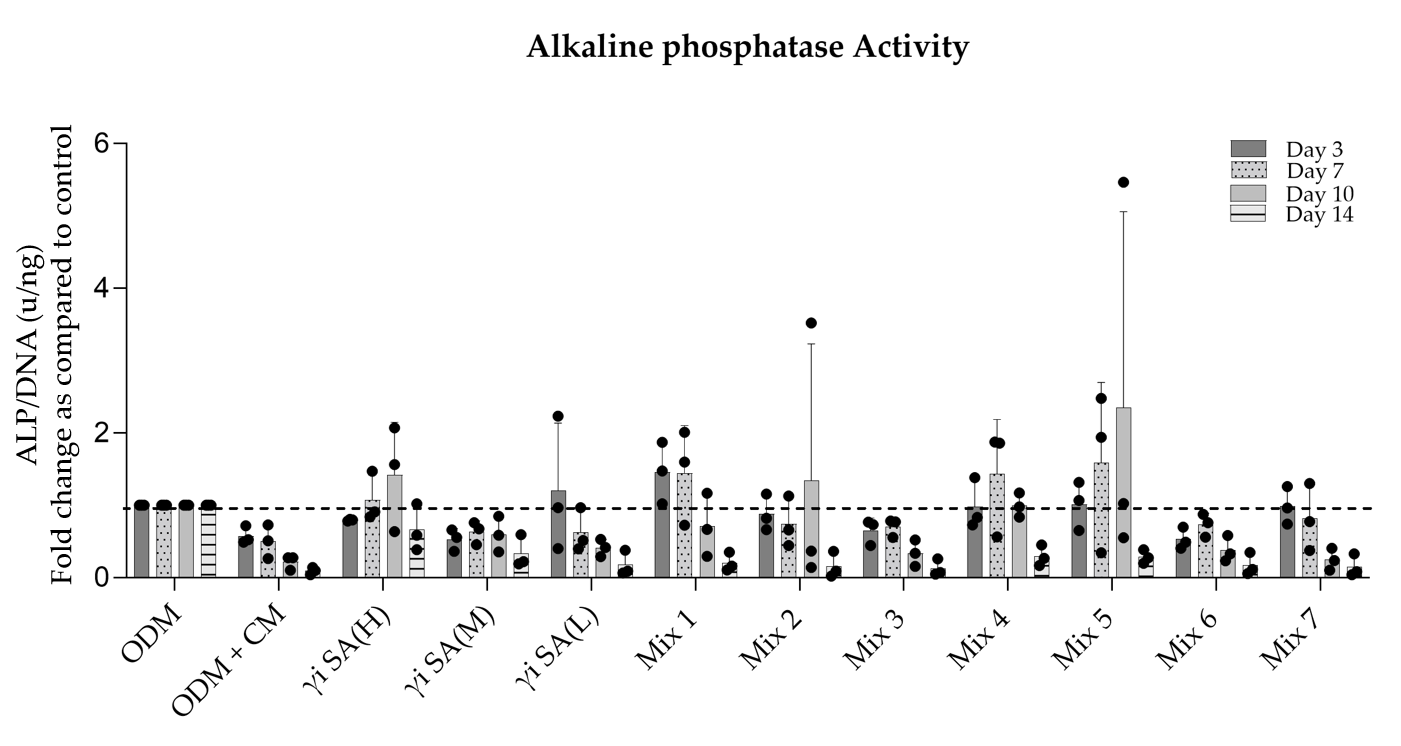


Supplementary Figure S4. Effect of indirect stimulation of γi *S. aureus* and mixtures on alkaline phosphatase levels in hMSC cultures, corrected for DNA on days 3, 7, 10, and 14. hMSC were cultured with conditioned medium obtained from hPBMCs (n = 6) stimulated with γi *S. aureus* in different concentrations and mixtures without osteogenic factor dexamethasone. All data were normalized to the osteogenic medium (ODM) control wells. The CM was added in the ratio of 1: 4 (CM: ODM). The graph represents the mean ± SD of technical triplicates performed per donor for three individual MSC donors.

Supplementary Figure S5.


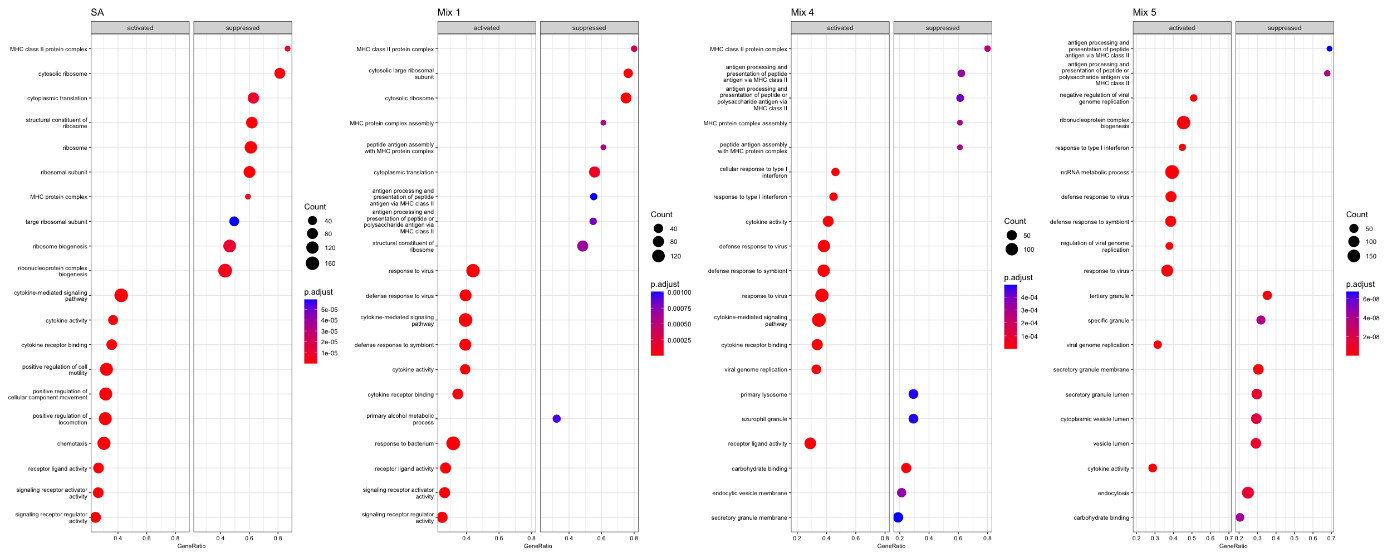


Supplementary Figure S5. The bubble plot displaying the top 10 GO terms upregulated in gene set enrichment analysis compared pre-defined GO resource for γi *S. aureus* and mixtures (Mix1, Mix 4, and Mix5) stimulated hPBMCs as compared to the unstimulated control.

Supplementary Figure S6.


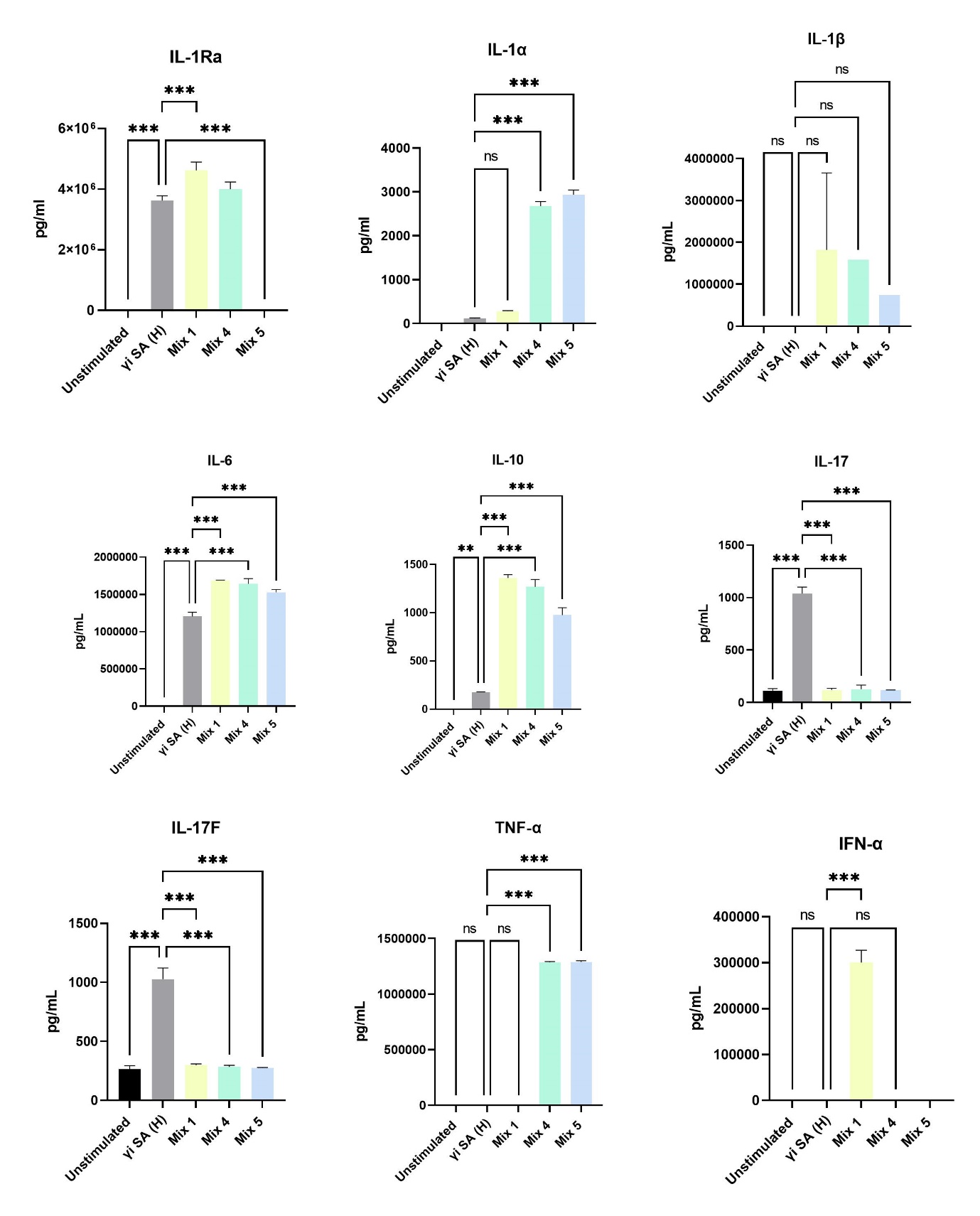


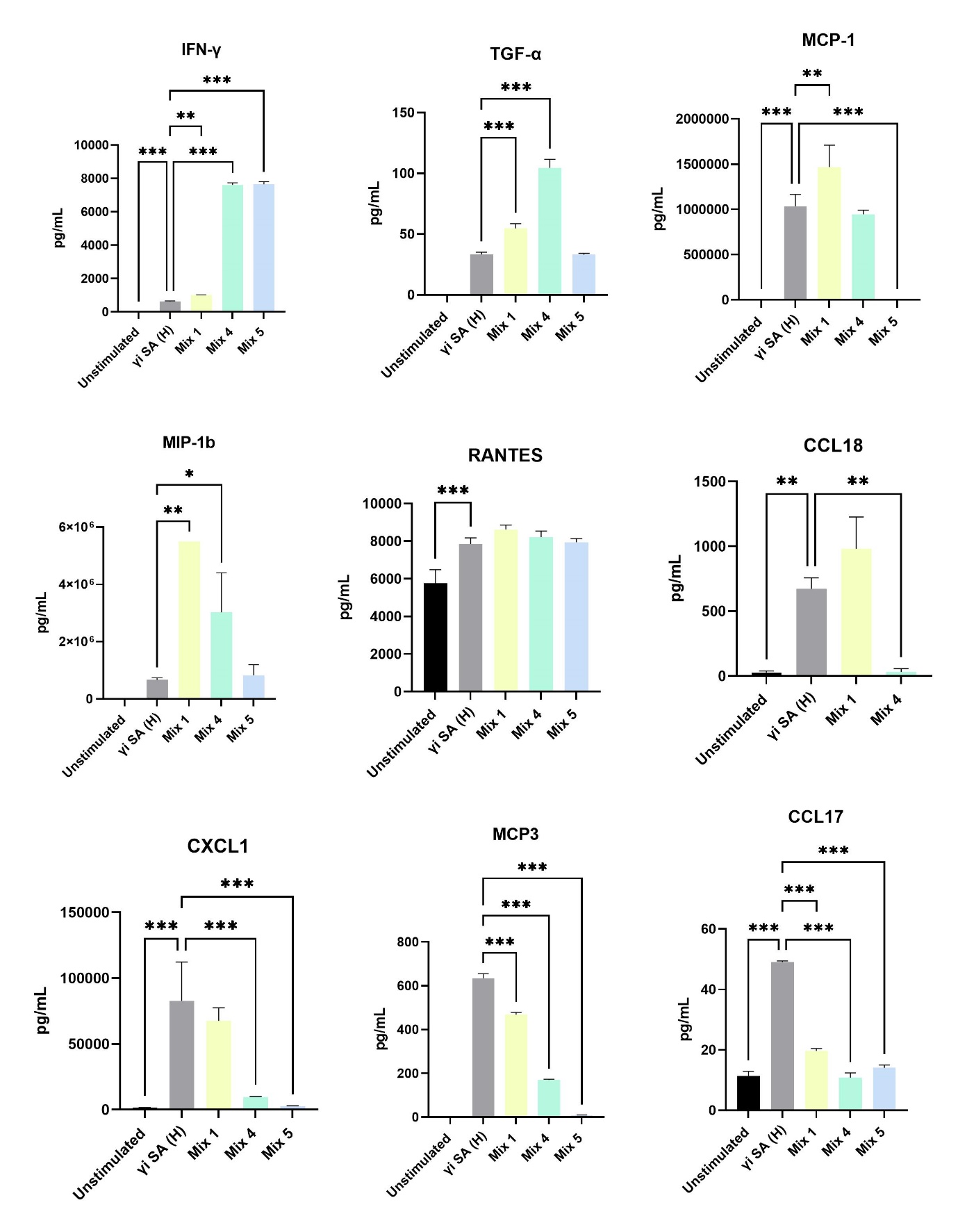


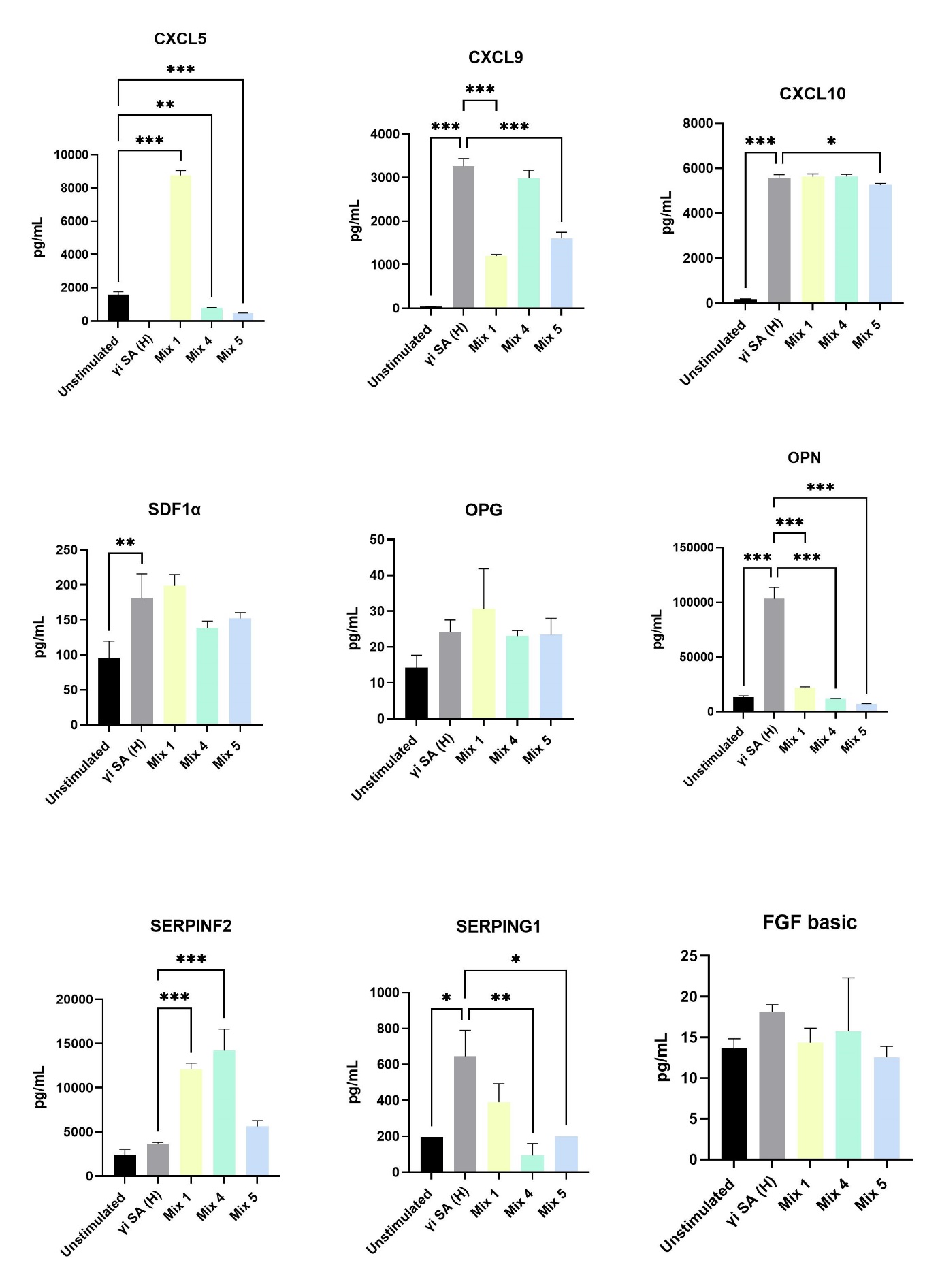


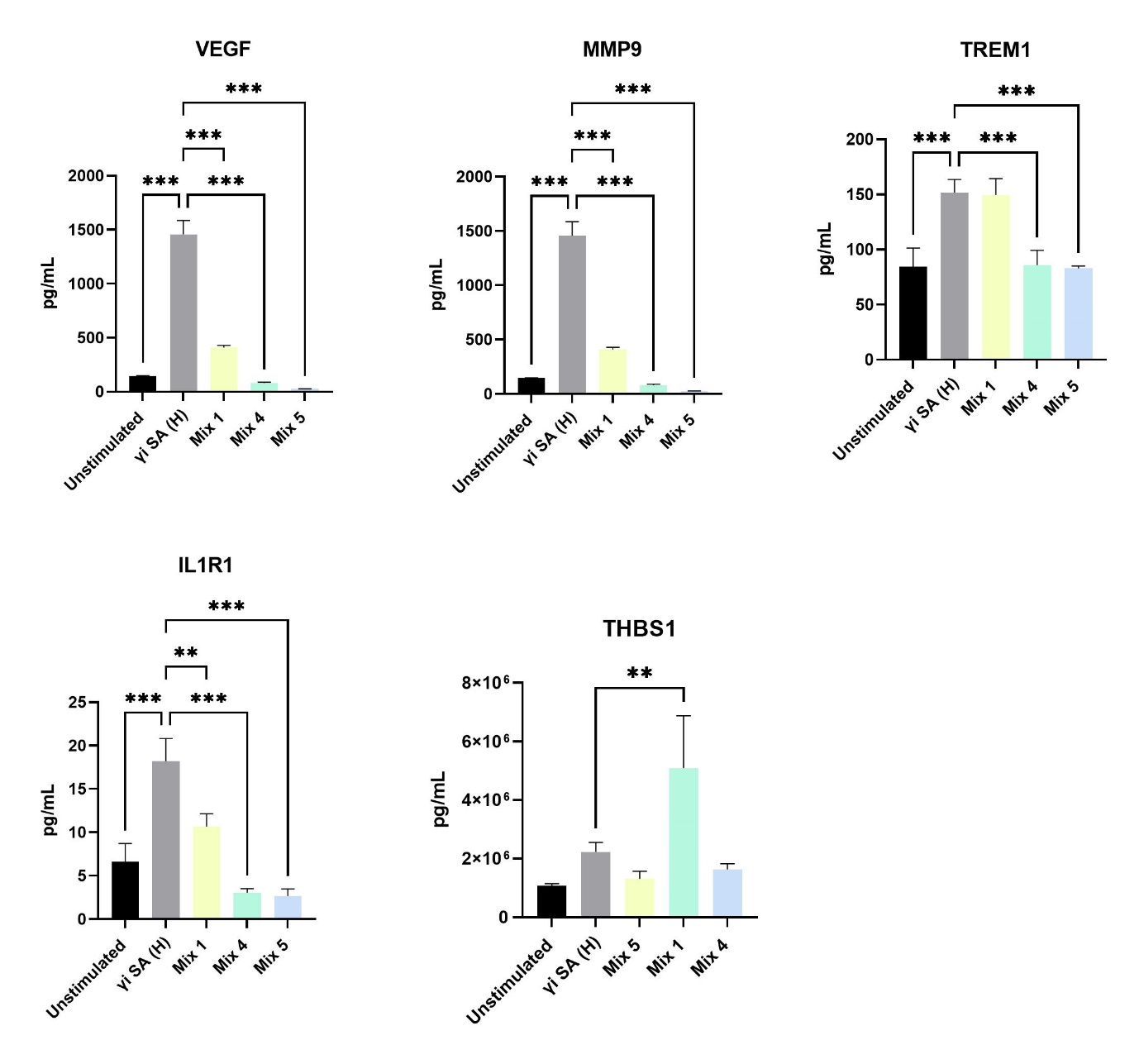


Supplementary Figure S6. Concentrations in the CM for selected proteins from figure 5. (a) hPBMCs (n=9) were stimulated with either γi *S. aureus* or mixtures for 24 hours. The conditioned medium obtained from the stimulation was pooled and used to characterize its composition using Luminex assay. All the groups were normalized to the unstimulated hPBMCs as the control. Data are presented in mean + standard deviation. Significance was tested using one-way ANOVA with post hoc Sidak’s test for multiple comparisons. * *p* < 0.05, ** *p* < 0.01, *** *p* < 0.001.
